# Anti-Inflammatory Effects of *Artemisia* Leaf Extract in Mice with Contact Dermatitis *In Vitro* and *In Vivo*

**DOI:** 10.1155/2016/8027537

**Published:** 2016-08-28

**Authors:** Chanyong Yun, Youngchul Jung, Wonjoo Chun, Beodeul Yang, Junghyun Ryu, Chiyeon Lim, Jung-Hoon Kim, Hyungwoo Kim, Su-In Cho

**Affiliations:** ^1^Division of Pharmacology, School of Korean Medicine, Pusan National University, Busan, Republic of Korea; ^2^College of Medicine, Dongguk University, Goyang-si, Gyeonggi-do 10326, Republic of Korea

## Abstract

The leaves of* Artemisia argyi* Lev. et Vant. and* A. princeps* Pamp. are well known medicinal herbs used to treat patients in China, Japan, and Korea with skin problems such as eczema and itching, as well as abdominal pain and dysmenorrhoea. We investigated the anti-inflammatory effects of* Artemisia* leaf extract (ALE) using CD mice and Raw 264.7 cells. The effects of ALE on histopathological changes and cytokine production in ear tissues were assessed in mice with CD induced by 1-fluoro-2,4-dinitrobenzene (DNFB). Moreover, the anti-inflammatory effects on production levels of prostaglandin E_2_ (PGE_2_) and nitric oxide (NO) and expression levels of cyclooxygenase 2 (COX-2) and inducible nitric oxide synthase (iNOS) were investigated in Raw 264.7 cells. Topical application of ALE effectively prevented ear swelling induced by repeated DNFB application. ALE prevented epidermal hyperplasia and infiltration of immune cells and lowered the production of interferon- (IFN-) gamma (*γ*), tumour necrosis factor- (TNF-) alpha (*α*), and interleukin- (IL-) 6 in inflamed tissues. In addition, ALE inhibited expression of COX-2 and iNOS and production of NO and PGE_2_ in Raw 264.7 cells. These results indicate that* Artemisia* leaf can be used as a therapeutic agent for inflammatory skin diseases and that its anti-inflammatory effects are closely related to the inhibition of inflammatory mediator release from macrophages and inflammatory cytokine production in inflamed tissues.

## 1. Introduction

 Contact dermatitis (CD) in the framework of occupational diseases remains prevalent among workers worldwide, impacting their quality of life and workability. The employees most affected by CD are hairdressers, healthcare workers, and metal workers [1] because they are continuously exposed to harmful environments when working. As a result, employees with CD tend to use anti-inflammatory and immunomodulatory agents such as corticosteroids repeatedly [2]. Corticosteroids are effective and powerful agents for CD, but their doses should be restricted because of their adverse side effects. Herbal medicines have recently emerged in the framework of complementary and alternative medicines (CAM) for corticosteroids because they have relatively lower cost and safety [3].

Herbs belonging to the* Artemisia* genus are widely used as medicine worldwide. The leaves of* Artemisia argyi* Lev. et Vant. and* A. princeps* Pamp. are frequently used as traditional or folk medicines for patients with abdominal pain, dysmenorrhoea, uterine haemorrhage, and inflammation in China, Japan, and Korea [4].* A. argyi,* Chinese mugwort, is herbaceous perennial plant known in Japanese as* gaiyou* and in Chinese as* aiye*.* A. princeps,* Japanese mugwort, is a perennial and very vigorous plant known as* yomogi* in Japanese. Recently, the leaves of* A. argyi* and* A. princeps* and their components have been shown to have antitumour [5–8], antifungal [9], anticoagulant [10], antidiabetic [11], and anti-inflammatory [12, 13] activity.

Based on these findings, we examined the effects of* Artemisia* leaf extract (ALE) on inflamed tissues in mice with CD and anti-inflammatory activities in Raw 264.7 cells. Specifically, the effects of ALE on histopathological changes including ear swelling, epidermal hyperplasia, immune cell infiltration, and cytokine production such as interferon- (IFN-) gamma (*γ*), tumour necrosis factor- (TNF-) alpha (*α*), interleukin- (IL-) 6, and IL-10 in ear tissues were assessed in mice with CD induced by topical application of 1-fluoro-2,4-dinitrobenzene (DNFB). The anti-inflammatory effects on production levels of prostaglandin E_2_ (PGE_2_) and nitric oxide (NO) and the expression levels of cyclooxygenase 2 (COX-2) and inducible nitric oxide synthase (iNOS) were investigated in Raw 264.7 cells.

## 2. Materials and Methods

### 2.1. Preparation of ALE

Artemisia leaf was purchased from Gwangmyungdang (Ulsan, Korea). The* Artemisia* leaf consisted of a mixture of* A. argyi* and* A. princeps* and was authenticated by Professor Jung-Hoon Kim, one of the authors of this study. Twenty grams of* Artemisia* leaf was immersed in 500 mL of methyl alcohol and sonicated for 15 min, after which they were extracted for 24 h. Following extraction, the supernatant was transferred and the* Artemisia* leaf was again extracted with 500 mL of methanol for 24 h. The two extracts were then combined and filtered through Whatman no. 20 filter paper, after which they were condensed using a rotary evaporator (EYELA, Tokyo, Japan). The evaporated extract was subsequently dried using a lyophilizer (Labconco, Kansas City, MO, USA), which yielded 1.04 g of freeze-dried powder (yield, 5.21%). Specimens of crude material and* Artemisia* leaf methanol extract (ALE, Voucher no. MH2013-040) were deposited in the herbarium located in the School of Korean Medicine, Pusan National University.

### 2.2. Animals

Six-week-old male Balb/c mice were obtained from Samtako (Incheon, Korea). All mice used in this experiment were housed in the cages under specific conditions, including a 12 h light/dark cycle and specific pathogen-free conditions. In addition, mice were provided with free access to standard rodent feed and water. We conducted all animal experiments according to institutional guidelines and all experimental procedures were approved by our animal care committee (PNU-2012-0140).

### 2.3. CD Induction and Experimental Schedule

**Figure 1:**
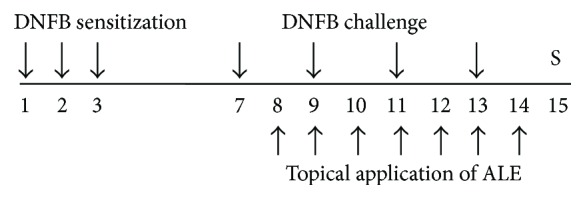
Experimental schedule. Mice in all experimental groups were sensitized by DNFB for three successive days and then challenged every other day (four times). The ALE group was topically treated with 30 or 300 *μ*g/ear of ALE (*n* = 8). The DEX group was topically treated with 75 *μ*g/ear of DEX for seven days from day 8 to day 14 (*n* = 6). All animals were sacrificed on day 15. S indicates sacrifice.

CD was induced using our standard method as previously described [14]. Briefly, 0.1% DNFB (50 *μ*L) in vehicle composed of acetone and olive oil (4 : 1, AOO) was applied onto the shaved back of mice for three successive days (sensitization). Mice were then treated by application of 0.2% DNFB (30 *μ*L) in vehicle onto the backside of their ears every two days. For topical treatment with drugs, dexamethasone (DEX) and ALE were dissolved in ethanol, filtered using a syringe filter (0.45 *μ*m), and finally diluted in vehicle (AOO : ethanol, 4 : 1). ALE (30 or 300 *μ*g/ear) was topically applied onto the backside of ears for 7 days. A total of 36 mice were used in this study (NOR group and DEX group, 6 mice; CTL group and ALE group, 8 mice). The experimental procedures are summarized in Figure 1.

### 2.4. Effects on Ear Thickness and Weight

Mice were sacrificed with CO_2_, after which ear pieces (5 mm in diameter) obtained via dermal punch were weighed using a microbalance and the thicknesses of both ears were measured with digimatic calipers (Mitutoyo, Kanagawa, Japan) at the same time.

### 2.5. Tissue Preparation and Staining

Obtained tissues were fixed in 4% formalin for 24 h and then dehydrated using ethyl alcohol. Next, all tissues were soaked in xylene and finally embedded in paraffin. Ear tissues (4 *μ*m) were subsequently resected, after which sections were stained with haematoxylin-eosin (H/E) and observed using a light microscope (50x).

### 2.6. Evaluation of Hyperplasia in the Epidermis and Infiltration of Immune Cells

To evaluate hyperplasia in the epidermis and infiltration of immune cells, five nonoverlapping fields per slide were randomly selected and captured with a light microscope. The height from the basal lamina to the top of the stratum granulosum was quantified to evaluate the epidermal thickness. Five lengths were used to calculate the mean epidermal thicknesses of each tissue slide. Total immune cell numbers were quantified by counting immune cells in the same size counting grid.

### 2.7. Measurement of Cytokine Production

Cytokine levels in ear tissues were evaluated using a mouse inflammation cytometric bead array (CBA) kit (BD Biosciences, San Jose, CA, USA). Briefly, to obtain tissue lysates, resected inflamed tissues were lysed using protein extraction solution (Intron Bio, Daejeon, Korea) and a homogenizer (Next Advance, NY, USA). Next, 50 *μ*g of lysates was used to evaluate the levels of TNF-*α*, IFN-*γ*, IL-6, and IL-10.

### 2.8. Cell Culture

Raw 264.7 cells, the immortalized murine macrophage cell line, were cultured using DMEM (HyClone, Logan, UT, USA) containing foetal bovine serum (10%, FBS) and antibiotics (1%, penicillin-streptomycin). Cells were maintained at 37°C under 5% CO_2_.

### 2.9. Determination of Nitric Oxide (NO) Production

Cells were seeded in 96-well plates at a density of 1 × 10^4^ cells/well and then incubated overnight. Next, cells were treated with the indicated concentrations of ALE for 4 h, after which they were stimulated with 1 *μ*g/mL of lipopolysaccharide (LPS) for 20 h. Following stimulation, 100 *μ*L of supernatants was mixed with 100 *μ*L of Griess Reagent (2% sulphanilamide in 10% H_3_PO_4_ and 0.2% of N-(1-naphthyl)ethylenediamine in distilled water) and then incubated at room temperature for 10 min. The absorbance at 540 nm was subsequently measured using a spectrophotometer (TECAN, Männedorf, Switzerland). The production levels of NO were determined using a NaNO_2_ serial dilution standard curve.

### 2.10. Measurement of Prostaglandin E2 (PGE_2_) Production

The production of PGE_2_ was measured by enzyme-linked immunosorbent assay (ELISA) using PGE_2_ ELISA assay kit (Enzo Life Science, Farmingdale, NY, USA). Briefly, cells were treated as previously described. The culture supernatants were then collected, after which the optical densities were measured using a spectrophotometer (TECAN, Männedorf, Switzerland) at a wavelength of 405 nm.

### 2.11. Western Blotting

The changes in protein expression following treatment were evaluated by western blot analysis. Briefly, ALE and LPS treated Raw 264.7 cells were harvested, lysed, and homogenized as previously described. The protein concentration of each cell lysate was then calculated using a bicinchoninic acid (BCA) assay. Primary antibodies against iNOS (482728, Merck Millipore, Darmstadt, Germany, 1:1000), COX-2 (sc-19999, Santa Cruz Biotechnology, Santa Cruz, CA, USA, 1:1000), *β*-actin (Santa Cruz Biotechnology, Santa Cruz, CA, USA), and horseradish-conjugated secondary antibody (Enzo Life Sciences, Farmingdale, NY, USA, 1:3000) were used to detect specific protein levels. In addition, a West-Q Chemiluminescent Substrate Kit (GenDEPOT, Barker, TX, USA) and a LAS 4000 mini (GE Healthcare, Piscataway, NJ, USA) were used to visualize the antigen-antibody complex.

### 2.12. Statistical Analysis

The Mann-Whitney* U* test was used for data obtained from* in vivo* experiments; Student's* t-*test was used for data obtained from* in vitro* experiments, and Prism 5 for window version 5.01 (GraphPad Software Inc., CA, USA) was used for all analyses. All data are presented as the means ± standard deviation. A *P* < 0.05 was considered significant.

## 3. Results

### 3.1. ALE Prevented Ear Swelling Induced by Repeated DNFB Application

**Figure 2:**
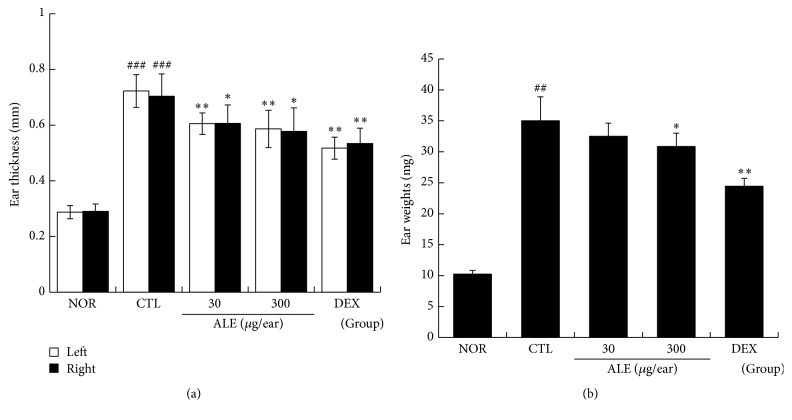
Effect of ALE on ear swelling in CD mice. The ear thickness and weight were measured on day 15. NOR: nontreated normal; CTL: nontreated CD; 30 or 300, 30, or 300 *μ*g/ear of ALE; DEX: 75 *μ*g/ear of DEX. (a) Ear thickness. (b) Ear weight. Values are presented as the means ± SD. ^##^ *P* < 0.01 and ^###^ *P* < 0.001 compared to the NOR group and ^*∗*^ *P* < 0.05 and ^*∗∗*^ *P* < 0.01 compared to the CTL group.

At the end of experiment, the thicknesses and weights of both ears were evaluated. In CTL group, repeated treatment with DNFB elevated the levels of ear thickness and weight more than three times relative to the normal group. 30 and 300 *μ*g/ear of ALE treatment effectively inhibited enlargement of ear thickness (Figure 2(a)) and 300 *μ*g/ear of ALE inhibited ear weight gain significantly (Figure 2(b)). DEX was more effective than ALE (Figure 2).

### 3.2. ALE Prevented Epidermal Hyperplasia and Infiltration of Immune Cells in Inflamed Tissues

**Figure 3:**
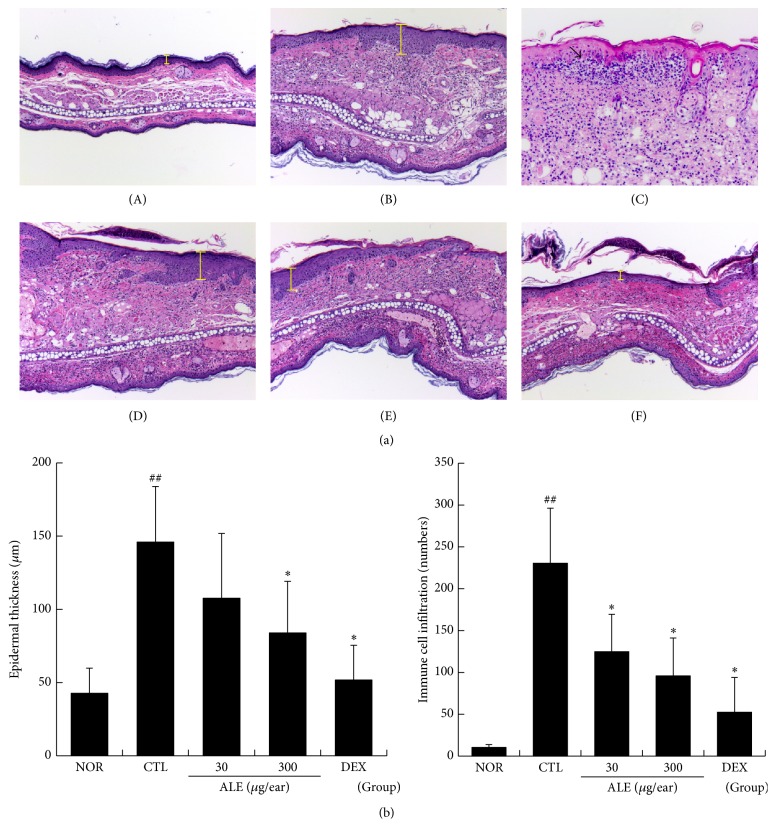
Effect of ALE on epidermal hyperplasia and infiltration of immune cells in CD mice. Skin tissue was observed under a light microscope. (A) NOR; ((B) and (C)) CTL; (D) 30 *μ*g/ear of ALE; (E) 300 *μ*g/ear of ALE; (F) 75 *μ*g/ear of DEX. Magnification, 50x. Yellow bars indicate the epidermis. Filled arrows indicate pustule areas (a). The epidermal thickness (A) and infiltration of immune cells (B) were evaluated using a quantitative method. Abbreviations are the same as in Figure 2. (b). Values are presented as the means ± SD. ^##^ *P* < 0.01 compared to the NOR group and ^*∗*^ *P* < 0.05 compared to the CTL group.

The effects of ALE on epidermal hyperplasia, one of the major features of CD, and immune cell infiltration were investigated. Marked increases in epidermal thickness (yellow bars) and infiltration of immune cells into inflamed tissues were observed in the nontreated CD mice. The main populations of infiltrated immune cells were neutrophil and macrophage. In addition, a large pustule (filled arrow) and vesicles were also observed (Figure 3(a)). Treatment with ALE effectively inhibited epidermal hyperplasia and reduced immune cell infiltration compared to the CTL mice (Figure 3(b)). DEX treatment was most effective among all experimental groups (Figure 3).

### 3.3. ALE Lowered the Production Levels of TNF-*α*, IFN-*γ*, and IL-6 in Inflamed Tissues

**Figure 4:**
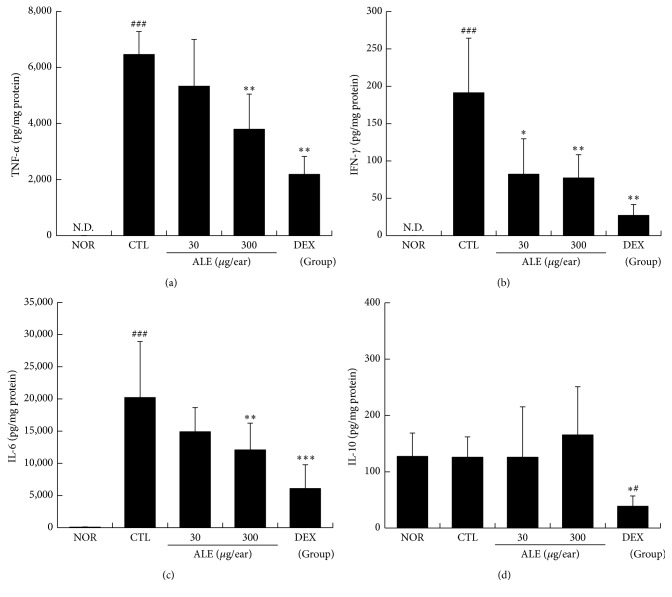
Effect of ALE on production levels of cytokines in inflamed tissues. The cytokine levels in inflamed tissues were evaluated. (a) TNF-*α*; (b) IFN-*γ*; (c) IL-6; (d) IL-10. N.D., not detected. Values are presented as the means ± SD. ^#^ *P* < 0.05 and ^###^ *P* < 0.001 compared to the NOR group and ^*∗*^ *P* < 0.05, ^*∗∗*^ *P* < 0.01, and ^*∗∗∗*^ *P* < 0.001 compared to the CTL group.

We also checked the effects of ALE on cytokine productions in inflamed tissues. In our results, elevated levels of TNF-*α*, IFN-*γ*, and IL-6 production were observed in CTL mice (Figure 4). These increases in inflammatory cytokines were significantly prevented by topical application of ALE. Treatment with 300 *μ*g/ear of ALE significantly lowered the production levels of TNF-*α*, IFN-*γ*, and IL-6, respectively. The IL-10 production levels were not affected by induction of CD or treatment with ALE, and only the DEX treated group showed lower production of IL-10 than the normal and control groups (Figure 4).

### 3.4. ALE Inhibited iNOS Expression and NO Production in Raw 264.7 Cells

**Figure 5:**
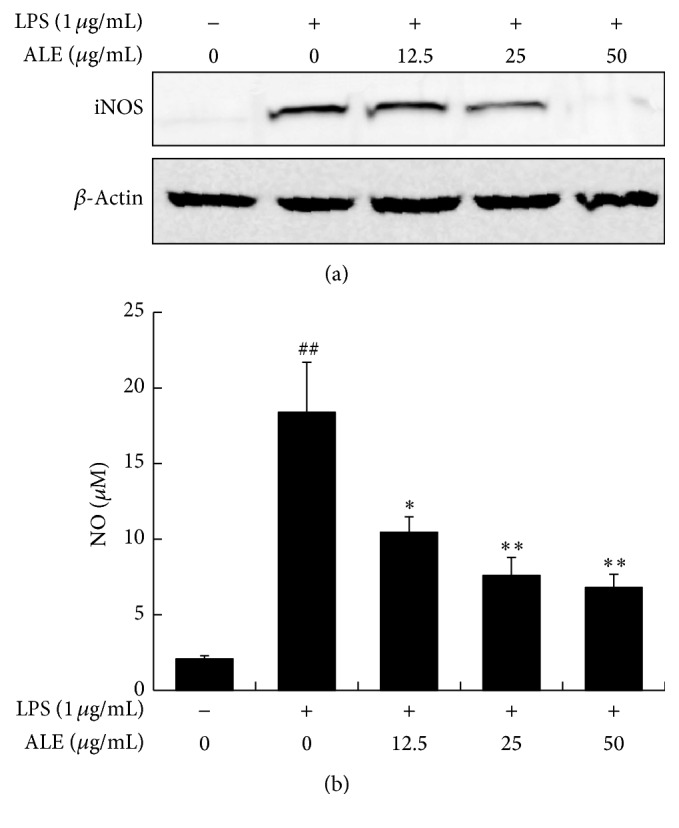
Effects of ALE on iNOS expression and NO production in Raw 264.7 cells. Cells were incubated with various concentrations of ALE for 4 h and then activated with 1 *μ*g/mL of LPS for 20 h. (a) iNOS expression; (b) NO production. Values are presented as the means ± SD. ^##^ *P* < 0.01 compared to the NOR group and ^*∗*^ *P* < 0.05 and ^*∗∗*^ *P* < 0.01 compared to the CTL group.

The inhibitory effects of ALE on iNOS expression induced by LPS were investigated. Expression of iNOS was elevated in the LPS treated CTL group compared to the normal group. Treatment with more than 25 *μ*g/mL of ALE lowered iNOS expression in a concentration dependent manner (Figure 5(a)). In addition, treatment with LPS elevated NO production level more than nine times relative to neither LPS nor ALE treated group. This increase in No production was effectively inhibited by ALE in a dose dependent fashion (Figure 5(b)).

### 3.5. ALE Inhibited COX-2 Expression and PGE_2_ Production in Raw 264.7 Cells

**Figure 6:**
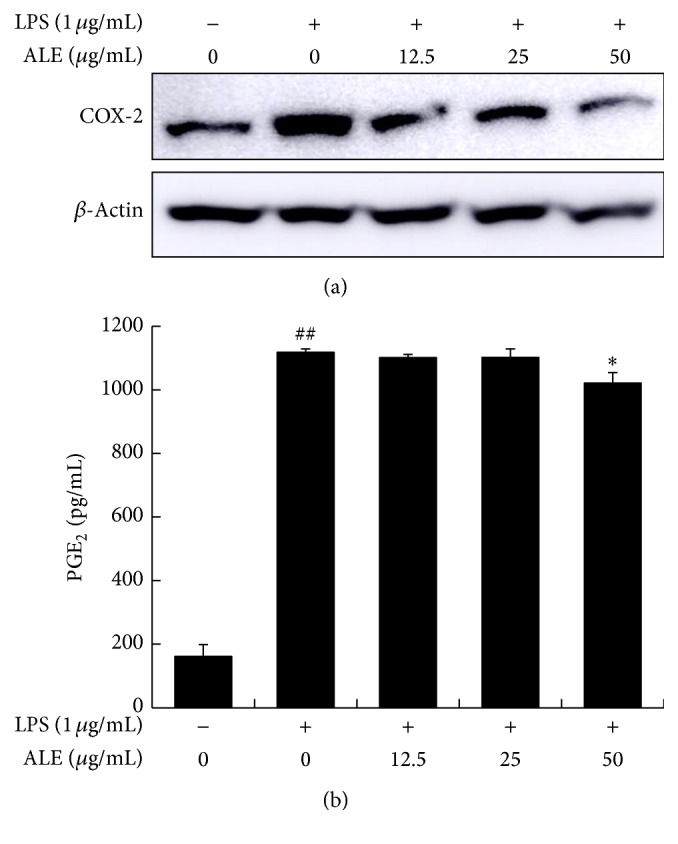
Effects of ALE on COX-2 expression and PGE_2_ production in Raw 264.7 cells. Cells were treated with various concentrations of ALE for 4 h and then activated with 1 *μ*g/mL of LPS for 20 h. The COX-2 expression levels were then detected by western blot analysis (a), while PGE_2_ production was measured by ELISA. Values represented are the means ± SD. ^##^ *P* < 0.01 compared to the NOR group and ^*∗*^ *P* < 0.05 compared to the CTL group (b).

The LPS stimulated Raw 264.7 cells showed marked increases in COX-2, representative COX enzyme, and treatment with ALE inhibited COX-2 expression (Figure 6(a)). In addition, the production of PGE_2_, one of the major final metabolites, was markedly induced by LPS stimulation. Treatment with 50 mg/mL of ALE inhibited PGE_2_ production significantly (Figure 6(b)).

## 4. Discussion

*Artemisia* species have traditionally been utilized for amelioration of diseases including malaria, hepatitis, parasites, and various cancers worldwide. The anti-inflammatory effects of* A. princeps* in antigen-stimulated T cells and regulatory T cells have been reported by Chang et al. [15]. Additionally, the major active components of* Artemisia* species are sesquiterpenoids and flavonoids such as eupatilin and jaceosidin, which are extracted from most herbs in* Artemisia* species, and these components have been reported to have anti-inflammatory effects in mice [16]. Based on the anti-inflammatory and immunomodulatory effects of* Artemisia* species, we investigated whether ALE can reduce inflammatory reactions in an animal model of CD to ameliorate its symptoms.

The skin of CD patients tends to thicken because of chronic and repeated inflammatory reactions, which are closely related to epidermal and dermal hyperplasia [17]. In our experiment, repeated application of DNFB induced ear swelling, which was effectively inhibited by topical application of ALE (Figure 2). Given that the degree of ear swelling is recognized as an index of inflammatory reaction, these results imply that ALE can exert anti-inflammatory action in animal models of CD.

In our animal model of CD, repeated application of DNFB induced hyperplasia in the epidermis and massive infiltration of immune cells to the epidermis and into the connective tissues. In addition, spongiotic changes, vesicles, and pustules were seen in the control group (Figure 3(a)(B)). Topical application of ALE effectively inhibited hyperplasia in the epidermis and infiltration of immune cells. In addition, the area of spongiotic changes and vesicles was diminished and pustules were rarely seen in the ALE and DEX group (Figure 3). These findings indicate that ALE can act as an anti-inflammatory agent in CD to reduce immune cell infiltration, resulting in inhibition of spongiotic changes, as well as pustule and vesicle formation.

TNF-*α* and IFN-*γ*, hallmarks of Th1 skewing reaction, can stimulate keratinocytes, which subsequently proliferate, resulting in epidermal hyperplasia [18]. These two cytokines are also closely related to infiltration of immune cells into the inflamed tissues. In the inflammatory cascade of CD, activated keratinocytes can release TNF-*α* and IL-6, which are growth promoting cytokines, resulting in accelerated immune cell infiltration and prolonged lifespan of immune cells [18]. IL-6 has also been reported to accelerate proliferation and migration of keratinocytes, leading to skin disorders, including psoriasis [19]. In this study, topical treatment with ALE effectively reduced production levels of TNF-*α*, IFN-*γ*, and IL-6 in ear tissues (Figure 4). When combined with previous results, these findings imply that ALE can inhibit hyperplasia in the epidermis and infiltration of immune cells via regulation of proinflammatory cytokines, finally leading to inhibition of enlargement of skin thickness.

Histopathological analyses revealed that many types of inflammatory cells, including macrophages, infiltrate into the dermis and epidermis in CD. Macrophages also play an important role by releasing inflammatory mediators such as NO, prostaglandins, and cytokines during initiation of CD. For these reasons, we also investigated anti-inflammatory effects of ALE* in vitro* using a macrophage cell line, Raw 264.7. Treatment with up to 100 *μ*g/mL of ALE had no effect on the viability of Raw 264.7 cells (data not shown). ALE significantly inhibited iNOS expression and NO production in a dose dependent manner (Figure 5). In addition, ALE inhibited COX-2 expression and PGE_2_ production induced by LPS stimulation (Figure 6). These findings indicate that ALE inhibits inflammatory response in macrophages via regulation of NO and PGE_2_ production. As shown in Figures 5 and 6, ALE strongly inhibited iNOS expression and NO production, while it had little effect on PGE_2_ production or COX-2 expression. This difference in efficacy may indicate that ALE can affect other subtypes of prostaglandin.

It is well known that intracellular signaling pathways such as Erk, JNK, and p38 and transcription factors such as CREB and NF-*κ*B play a central role in activation and inflammatory mediator production in macrophages [20]. We checked the effects of ALE on expression levels of signaling pathways and the NF-*κ*B pathway. Treatment with ALE prevented phosphorylation of p38 but had no effect on the NF-*κ*B pathway (data not shown). Considering these results, the anti-inflammatory effects of ALE may be closely related to the p38 signaling pathway, and the NF-*κ*B pathway may not participate in the anti-inflammatory mechanisms of ALE.

Kim et al. reported immune-stimulatory effects of hot water extracts of the leaves of* A. princeps* [21]. Specifically, they found that extracts of* A. princeps* elevated production levels of NO and TNF-*α*, which is inconsistent with our results. These differences may be attributed to different extraction methods. Many previous studies of* A. princeps* have shown anti-inflammatory or antiallergic effects, as well as antioxidative effects. In addition, components isolated from* A. princeps* such as eupatilin and jaceosidin have been reported to have inhibitory effects against IgE-induced hypersensitivity and carrageenan-induced inflammation, respectively [16, 22]. However, Kim et al. did not describe why their findings were inconsistent with those of previous results. In addition, water extracts of plant materials should also be checked for microbial contamination before use because they are more easily contaminated than methanol or ethanol extracts.

Overall, the results of this study suggest that the anti-inflammatory effects of ALE in CD mice are closely related to regulation of the activation of immune cells, especially macrophages and cytokine production in inflamed tissues.

## 5. Conclusion

In this study, ALE inhibited the release of NO and PGE_2_ in macrophages. In addition, ALE effectively lowered the production levels of TNF-*α*, IFN-*γ*, and IL-6 in inflamed tissues. These anti-inflammatory actions prevented epidermal hyperplasia and immune cell infiltration. Finally, ALE effectively inhibited ear swelling induced by DNFB. These results imply that ALE can be used for the treatment of patients with CD as a complement and alternative medicine (CAM) to corticosteroids.

## References

[1] Diepgen T. L., Kuss O., Blesius C. R., Schmidt A., Diepgen T. L. (2001). Occupational skin diseases in Northern Bavaria between 1990 and 1999: a population-based study. *British Journal of Dermatology*.

[2] Cohen D. E., Heidary N. (2004). Treatment of irritant and allergic contact dermatitis. *Dermatologic Therapy*.

[3] Wen M.-C., Wei C.-H., Hu Z.-Q. (2005). Efficacy and tolerability of antiasthma herbal medicine intervention in adult patients with moderate-severe allergic asthma. *The Journal of Allergy and Clinical Immunology*.

[4] Kim C. M., Shin M. G., An D. G., Lee K. S. (1997). *The Encyclopedia of Oriental Herbal Medicine*.

[5] Bao X., Yuan H., Wang C., Liu J., Lan M. (2013). Antitumor and immunomodulatory activities of a polysaccharide from *Artemisia argyi*. *Carbohydrate Polymers*.

[6] Choi E.-J., Kim G.-H. (2013). Antioxidant and anticancer activity of *Artemisia princeps* var. orientalis extract in HepG2 and Hep3B hepatocellular carcinoma cells. *Chinese Journal of Cancer Research*.

[7] Khan M., Yu B., Rasul A. (2012). Jaceosidin induces apoptosis in U87 glioblastoma cells through G2/M phase arrest. *Evidence-Based Complementary and Alternative Medicine*.

[8] Kim J.-H., Jung S.-H., Yang Y.-I. (2013). Artemisia leaf extract induces apoptosis in human endometriotic cells through regulation of the p38 and NF*κ*B pathways. *Journal of Ethnopharmacology*.

[9] Wenqiang G., Shufen L., Ruixiang Y., Yanfeng H. (2006). Comparison of composition and antifungal activity of *Artemisia argyi* Lévl. et Vant inflorescence essential oil extracted by hydrodistillation and supercritical carbon dioxide. *Natural Product Research*.

[10] Ryu R., Jung U. J., Kim H.-J. (2013). Anticoagulant and antiplatelet activities of artemisia princeps Pampanini and its bioactive components. *Preventive Nutrition and Food Science*.

[11] Yamamoto N., Kanemoto Y., Ueda M., Kawasaki K., Fukuda I., Ashida H. (2011). Anti-obesity and anti-diabetic effects of ethanol extract of *Artemisia princeps* in C57BL/6 mice fed a high-fat diet. *Food and Function*.

[12] Wang S., Li J., Sun J. (2013). NO inhibitory guaianolide-derived terpenoids from *Artemisia argyi*. *Fitoterapia*.

[13] Chung K.-S., Choi H.-E., Shin J.-S. (2015). Chemopreventive effects of standardized ethanol extract from the aerial parts of *Artemisia princeps* Pampanini cv. Sajabal via NF-*κ*B inactivation on colitis-associated colon tumorigenesis in mice. *Food and Chemical Toxicology*.

[14] Han H.-Y., Ryu M. H., Lee G. (2015). Effects of *Dictamnus dasycarpus* Turcz., root bark on ICAM-1 expression and chemokine productions in vivo and vitro study. *Journal of Ethnopharmacology*.

[15] Chang S. H., Jung E. J., Park Y. H. (2009). Anti-inflammatory effects of Artemisia princeps in antigen-stimulated T cells and regulatory T cells. *The Journal of Pharmacy and Pharmacology*.

[16] Min S.-W., Kim N.-J., Baek N.-I., Kim D.-H. (2009). Inhibitory effect of eupatilin and jaceosidin isolated from *Artemisia princeps* on carrageenan-induced inflammation in mice. *Journal of Ethnopharmacology*.

[17] Serup J. (1992). Characterization of contact dermatitis and atopy using bioengineering techniques. A survey. *Acta Dermato-Venereologica*.

[18] Corsini E., Galli C. L. (2000). Epidermal cytokines in experimental contact dermatitis. *Toxicology*.

[19] Kawakami M., Kaneko N., Anada H., Terai C., Okada Y. (1997). Measurement of interleukin-6, interleukin-10, and tumor necrosis factor- alpha levels in tissues and plasma after thermal injury in mice. *Surgery*.

[20] Bode J. G., Ehlting C., Häussinger D. (2012). The macrophage response towards LPS and its control through the p38 MAPK-STAT3 axis. *Cellular Signalling*.

[21] Kim T.-H., Lee S.-J., Rim H.-K. (2013). In vitro and in vivo immunostimulatory effects of hot water extracts from the leaves of *Artemisia* princeps* Pampanini* cv. Sajabal. *Journal of Ethnopharmacology*.

[22] Lee S. H., Bae E.-A., Park E.-K. (2007). Inhibitory effect of eupatilin and jaceosidin isolated from *Artemisia princeps* in IgE-induced hypersensitivity. *International Immunopharmacology*.

